# Bologna guidelines for diagnosis and management of adhesive small bowel obstruction (ASBO): 2017 update of the evidence-based guidelines from the world society of emergency surgery ASBO working group

**DOI:** 10.1186/s13017-018-0185-2

**Published:** 2018-06-19

**Authors:** Richard P. G. ten Broek, Pepijn Krielen, Salomone Di Saverio, Federico Coccolini, Walter L. Biffl, Luca Ansaloni, George C. Velmahos, Massimo Sartelli, Gustavo P. Fraga, Michael D. Kelly, Frederick A. Moore, Andrew B. Peitzman, Ari Leppaniemi, Ernest E. Moore, Johannes Jeekel, Yoram Kluger, Michael Sugrue, Zsolt J. Balogh, Cino Bendinelli, Ian Civil, Raul Coimbra, Mark De Moya, Paula Ferrada, Kenji Inaba, Rao Ivatury, Rifat Latifi, Jeffry L. Kashuk, Andrew W. Kirkpatrick, Ron Maier, Sandro Rizoli, Boris Sakakushev, Thomas Scalea, Kjetil Søreide, Dieter Weber, Imtiaz Wani, Fikri M. Abu-Zidan, Nicola De’Angelis, Frank Piscioneri, Joseph M. Galante, Fausto Catena, Harry van Goor

**Affiliations:** 1 Department of Surgery, Radboud University Medical Center, Nijmegen, The Netherlands; 2 Addenbrooke’s Hospital, Cambridge, UK; 3 General Emergency and Trauma Surgery, Bufalini hospital, Cesena, Italy; 4 Acute Care Surgery, The Queen’s Medical Center, Honolulu, Hawaii USA; 5 Department of Trauma, Emergency Surgery and Surgical Critical Care, Massachusetts General Hospital, Boston, MA USA; 6 Department of Surgery, Macerata Hospital, Macerata, Italy; 7 Faculdade de Ciências Médicas (FCM), Unicamp Campinas, São Paulo, Brazil; 8 Albury Hospital, Albury, NSW Australia; 9 University of Florida, Gainesville, USA; 10 Department of Surgery, Trauma and Surgical Services, University of Pittsburgh School of Medicine, Pittsburgh, USA; 11 Second Department of Surgery, Meilahti Hospital, Helsinki, Finland; 12 Trauma Surgery, Denver Health, Denver, CO USA; 13 Erasmus MC, Rotterdam, The Netherlands; 14 Division of General Surgery Rambam Health Care Campus Haifa, Haifa, Israel; 15 General Surgery Department, Letterkenny Hospital, Letterkenny, Ireland; 16 Department of Traumatology, John Hunter Hospital and University of Newcastle, Newcastle, NSW Australia; 17 John Hunter Hospital, New Lambton Heights, New Zealand; 18 Department of Vascular and Trauma Surgery, Auckland City Hospital, Auckland, New Zealand; 19 Department of Surgery, UC San Diego Health System, San Diego, USA; 20 Trauma, Acute Care Surgery Medical College of Wisconsin/Froedtert Trauma Center Milwaukee, Milwaukee, Wisconsin USA; 21 Virginia Commonwealth University, Richmond, VA USA; 22 Division of Trauma & Critical Care, LAC+USC Medical Center, University of Southern California, Los Angeles, CA USA; 23 Department of General Surgery, Westchester Medical Center, Westchester, NY USA; 24 Department of General Surgery, Assuta Medical Centers, Tel Aviv, Israel; 25 Department of Surgery, Foothills Medical Centre, Calgary, Canada; 26 Department of Surgery, Harborview Medical Centre, Seattle, USA; 27 Trauma & Acute Care Service, St Michael’s Hospital, Toronto, ON Canada; 28 Department of General Surgery, University of Medicine Plovdiv, Plovdiv, Bulgaria; 29 R Adams Crowley Shock Trauma Center, University of Maryland, Baltimore, USA; 30 Department of Gastrointestinal Surgery, Stavanger University Hospital, Stavanger, Norway; 31 Department of Clinical Medicine, University of Bergen, Bergen, Norway; 32 Department of General Surgery, Royal Perth Hospital, The University of Western Australia and The University of Newcastle, Perth, Australia; 33 Department of Surgery, Sheri-Kashmir Institute of Medical Sciences, Srinagar, India; 34 Department of Surgery, College of Medicine and Health Sciences, UAE University, Al-Ain, United Arab Emirates; 35 Unit of Digestive Surgery, HPB Surgery and Liver Transplant, Henri Mondor Hospital, Créteil, France; 36 Canberra Hospital, Canberra, Australia; 37 Trauma and Acute Care Surgery and Surgical Critical Care Trauma, Department of Surgery, University of California, Davis, USA; 38 Emergency and Trauma Surgery, Parma Maggiore hospital, Parma, Italy; 39 Department of Surgery, Radboud University Nijmegen Medical Centre, P.O. Box 9101, 6500 HB Nijmegen, The Netherlands

**Keywords:** Small bowel obstruction, Adhesions, Surgery, Laparoscopy, Laparotomy

## Abstract

**Background:**

Adhesive small bowel obstruction (ASBO) is a common surgical emergency, causing high morbidity and even some mortality. The adhesions causing such bowel obstructions are typically the footprints of previous abdominal surgical procedures. The present paper presents a revised version of the Bologna guidelines to evidence-based diagnosis and treatment of ASBO. The working group has added paragraphs on prevention of ASBO and special patient groups.

**Methods:**

The guideline was written under the auspices of the World Society of Emergency Surgery by the ASBO working group. A systematic literature search was performed prior to the update of the guidelines to identify relevant new papers on epidemiology, diagnosis, and treatment of ASBO. Literature was critically appraised according to an evidence-based guideline development method. Final recommendations were approved by the workgroup, taking into account the level of evidence of the conclusion.

**Recommendations:**

Adhesion formation might be reduced by minimally invasive surgical techniques and the use of adhesion barriers. Non-operative treatment is effective in most patients with ASBO. Contraindications for non-operative treatment include peritonitis, strangulation, and ischemia. When the adhesive etiology of obstruction is unsure, or when contraindications for non-operative management might be present, CT is the diagnostic technique of choice. The principles of non-operative treatment are *nil* per os, naso-gastric, or long-tube decompression, and intravenous supplementation with fluids and electrolytes. When operative treatment is required, a laparoscopic approach may be beneficial for selected cases of simple ASBO.

Younger patients have a higher lifetime risk for recurrent ASBO and might therefore benefit from application of adhesion barriers as both primary and secondary prevention.

**Discussion:**

This guideline presents recommendations that can be used by surgeons who treat patients with ASBO. Scientific evidence for some aspects of ASBO management is scarce, in particular aspects relating to special patient groups. Results of a randomized trial of laparoscopic versus open surgery for ASBO are awaited.

## Background

Adhesive small bowel obstruction (ASBO) is one of the leading causes of surgical emergencies and in particular of surgical emergencies that require an emergent operations [1–4]. In the UK, small bowel obstruction was the indication for 51% of all emergency laparotomies [2]. Scott et al. reported on seven emergency surgical procedures that account for 80% of all general surgery emergency admissions, morbidity, deaths, and healthcare expenditures in the USA [3]. Adhesive small bowel obstruction was the most common diagnosis for both the top 2 (small bowel resection) and top 5 (adhesiolysis) procedures [3]. Post-operative adhesions are the leading cause of small bowel obstructions, accounting for 60% of cases [1].

ASBO causes considerable harm, resulting in 8 days of hospitalization on average and an in-hospital mortality rate of 3% per episode [5–8]. Between 20 and 30% of patients with adhesive small bowel obstruction require operative treatment [1, 9–11]. Length of hospitalization and morbidity depend on the need for surgical intervention. Average hospitalization after surgical treatment of ASBO is 16 days, compared to 5 days following non-operative treatment [12]. Associated costs in a Dutch study in 2016 were estimated at €16,305 for surgical and €2227 for non-operative treatment [12].

Although adhesive small bowel obstruction is a common condition, the prevention and treatment is often characterized by surgeons’ personal preferences rather than standardized evidence-based protocols. There is a large amount of conflicting and low-quality evidence in publications regarding treatment of adhesive small bowel obstruction.

Therefore, the World Society of Emergency Surgery (WSES) working group on ASBO has developed evidence-based guidelines to support clinical decision making in diagnosis and management of ASBO [11, 13]. In the present revision of these guidelines, all recommendations were updated according to the latest evidence available from the medical literature. Further, we have introduced two new sections: prevention of ASBO and special patient groups.

## Methods

The guideline was written under the auspices of the WSES by the ASBO working group. Systematic searches of the MEDLINE and Embase databases were carried out in October 2016 using the keywords relevant to each section. Terms relevant to each section of the guideline were mapped to MEDLINE Medical Subjects Headings (MeSH) terms, as well as searched for as text items. Articles describing randomized controlled trials and systematic reviews were searched for using the methodological filters of the Scottish Intercollegiate Guidelines Network (http://www.sign.ac.uk/methodological-principles.html). The bibliographies of included articles were subsequently hand-searched for other relevant references, and experts in the field were asked if they found any relevant reports missing.

### Critical appraisal

Articles selected to support recommendations were assessed using the levels of evidence as published by the Centre for Evidence-Based Medicine of the University of Oxford (www.cebm.net; Table 1). Articles were classified according to the type of article and individually assessed for methodological quality using the GRADE method as proposed by the GRADE working group. That working group has developed a common, sensible, and transparent approach to grading the quality of evidence and strength of recommendations (http://www.gradeworkinggroup.org). The main literature on which the conclusion for each relevant topic is based is stated with the conclusion, accompanied by the level of evidence (Table 2) [14, 15].

**Table 1:** Classification of evidence per article

Level of evidence	Interventional research	Studies concerning diagnostic accuracy	Studies on complications or side effects, etiology, prognosis
A1	Systematic review/meta-analysis of at least 2 independently performed level A2 studies		
A2	Double-blind controlled randomized comparative clinical trial of good study quality with an adequate number of study participants	Diagnostic test compared to reference test; criteria and outcomes defined in advance; assessment of test results by independent observers; independent interpretation of test results; adequate number of consecutive patients enrolled; all patients subjected to both tests	Prospective cohort with sufficient amount of study participants and follow-up, adequately controlled for confounders; selection in follow-up has been successfully excluded
B	Comparative studies, but without all the features mentioned for level A2 (including patient-control studies, cohort studies)	Diagnostic test compared to reference test, but without all the features mentioned in A2	Prospective cohort study, but without all the features mentioned for level A2 or retrospective cohort study or case-control study
C	Noncomparative studies		
D	Expert opinion

**Table 2:** Grading of the conclusions and recommendations according to the level of evidence and strength of recommendation

Level	Conclusion based on
A	Systematic review (A1) or at least 2 independent studies with evidence level A2 (“there is evidence that…”)
B	One study with evidence level A2 or at least 2 independent studies with evidence level B (“it is likely that…”)
C	One study with evidence level B or level C (“there are indications that…”)
D	Expert opinion (“the working group recommends…”)
Level	Recommendation
I	Strong recommendation
II	Weak recommendation (suggestion)

Conclusion and recommendations are graded according to the level of evidence from strong (“there is strong evidence for,” level A) to weak (“we cannot be confident,” level D). Recommendations were graded as strong recommendations (level I) or weak recommendation or suggestions (level II). Recommendations were considered strong recommendations if there is sufficient evidence (level A or B) demonstrating that the benefits of an intervention are of clinical importance and clearly outweigh the harm of the intervention. A concept guideline was sent to all involved for comment and approval after which internal consensus was reached between the members of the working group. Amendments were made based upon these comments, leading to the final version of this updated guideline.

### Definitions

#### Peritoneal adhesions

The term “peritoneal adhesions” or simply “adhesions” is defined as fibrous tissue that connects surfaces or organs within the peritoneal cavity that are normally separated. Such adhesions are the results of a pathological healing response of the peritoneum upon injury, as opposed to the normal “ad integrum” repair [16]. Typical adhesions form after peritoneal injury from abdominal surgery. Other conditions that may cause peritoneal injury resulting in adhesion formation include radiotherapy, endometriosis, inflammation, and local response to tumors. Adhesions from a non-operative etiology are often part of a more complex pathology that can cause chronic pain and complications as the result of adhesions and other mechanisms [17]. Management of chronic abdominal complications by adhesiolysis is controversial [18, 19]. The scope of the present guideline is limited to diagnosis and management of acute bowel obstructions.

#### Adhesive small bowel obstruction

Small bowel obstruction is a surgical emergency in which the obstruction of the small intestine hinders passage of intestinal contents. Small bowel obstruction is characterized by abdominal pain, vomiting, distention, and constipation. Adhesions are the single most common cause for small bowel obstruction [1, 20]. Nonadhesive etiologies of bowel obstruction include incarcerated hernias, obstructive lesions (malignant and benign), and a number of infrequent causes for bowel obstruction such as bezoars, inflammatory bowel disease, and volvulus [21–25]. Definitive confirmation of the adhesive etiology of bowel obstruction is made during operative treatment. Methods to confirm the adhesive etiology of bowel obstruction non-invasively include a history of previous episodes of bowel obstruction by adhesions or exclusion of other causes of bowel obstruction by imaging (often CT scan).

#### Adhesiolysis

Adhesiolysis refers to releasing adhesions either by blunt or sharp dissection during surgery. It can be the primary indication for an operation, as in a reoperation for small bowel obstruction caused by adhesions. Adhesiolysis is also performed during reoperations for indications not related to adhesions in order to obtain sufficient access to the operative field. Complicated adhesiolysis refers to the event of inadvertent injury while performing adhesiolysis. Injuries during adhesiolysis are most frequently made to the bowel. These bowel injuries are classified as:

- Seromuscular injury: injury to the visceral peritoneum (serosa) and smooth muscle layer of the bowel. The lumen of the bowel or leakage of bowel contents is not visible.
- Enterotomy: a full thickness injury to the bowel. The mucous layer or lumen of the bowel is visible, or there may be leakage of intestinal contents.
- Delayed diagnosed perforation: bowel injuries made during surgery that initially go unrecognized. Typically, the abdomen is closed at the end of procedure with the bowel injury still in place, causing patients to deteriorate during the postoperative course.

## Results

### Epidemiology

The risk of SBO is highest following colorectal, oncologic gynecological, or pediatric surgery [1, 26–28]. One in ten patients develops at least one episode of SBO within 3 years after colectomy [7]. Reoperations for ASBO occur in between 4.2 and 12.6% of patients after pediatric surgery patients, and 3.2% of colorectal patients [1, 29]. Recurrence of ASBO is also frequent; 12% of non-operatively treated patients are readmitted within 1 year, rising to 20% after 5 years. The risk of recurrence is slightly lower after operative treatment: 8% after 1 year and 16% after 5 years [30].

### Classification of adhesions

The most frequently used classification of adhesions in general surgery is the adhesion score according to Zühlke et al. (Table 3) [31]. The score is based on the tenacity and some morphologic aspects of the adhesions. The merits of this score are that it is easy to use and classifications are self-explanatory to most surgeons and gynecologists. The major drawback to the score is that it does not measure the extent of adhesions and that tenacity of adhesions can vary between different parts of the abdomen. The most used grading system in gynecological surgery is the American Fertility Society (AFS) score [32]. The score is designed for grading adhesions in the small pelvis. Adhesions are scored for extent and severity at four sites: right ovary, right tube, left ovary, and left tube. The scores for the right and left side are summed, and the final AFS score is the score for the side with the lowest summed score while discarding the score for the other side. Thus, a patient with an AFS score of 0 can still have adhesions. Further critiques for this score include a relatively low inter-observer reproducibility [33]. A modified AFS has therefore gained popularity in more recent studies [34].

**Table 3:** Classification of adhesions according to Zühlke et al.

Grade 0	No adhesions or insignificant adhesions
Grade 1	Adhesions that are filmy and easy to separate by blunt dissection
Grade 2	Adhesions where blunt dissection is possible but some sharp dissection necessary, beginning vascularization
Grade 3	Lysis of adhesions possible by sharp dissection only, clear vascularization
Grade 4	Lysis of adhesions possible by sharp dissection only, organs strongly attached with severe adhesions, damage of organs hardly preventable

**Fig. 1:**
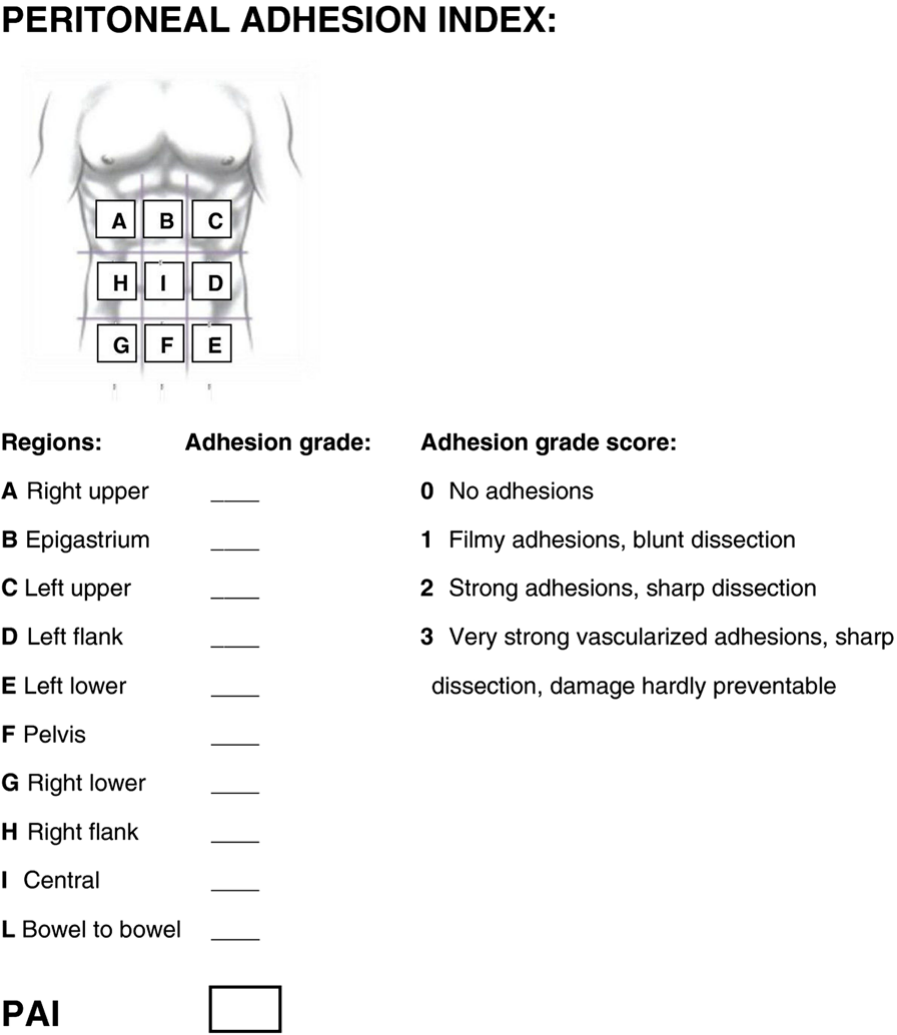
Peritoneal adhesion index. Reproduced with permission from [35]

A recently introduced score by the ASBO working group is the peritoneal adhesion index (PAI), which measures tenacity on a 1–3 scale at 10 predefined sites, to integrate tenacity and extent of adhesions in a single score (Fig. 1) [35]. This score is the only score that has been validated to be prognostic for convalescence after surgery for ASBO and the risk of injuries during adhesiolysis [36]. A limitation to all these adhesion scores is that they are only applicable to operative cases because they require operative assessment. Furthermore, none of them has yet been validated to correlate with the long-term risk for (recurrence of) adhesion-related complications.

A different type of classification in the field of ASBO is risk stratification that predicts the need for surgery. Zielinski reported on three radiological and clinical signs that correlate with the need for surgical exploration: mesenteric edema, absence of the small-bowel feces sign, and obstipation. The score was validated in 100 cases of ASBO and predicted the risk with a concordance index of 0.77 [37]. A more accurate model was reported by Baghdadi et al. This score comprises radiological findings, sepsis criteria, and comorbidity index. Although the score is somewhat complex to assess, it correlates with an area under the curve of 0.80 in a validation study of 351 cases [38].

### Prevention

#### Surgical technique

The main principles of prevention of adhesion and related complications are minimizing surgical trauma and the use of adjuvants to reduce adhesion formation. Laparoscopy is often believed to reduce adhesion formation and the risk for ASBO. In a systematic review of cohort studies, the incidence of reoperation for ASBO was 1.4 (95% CI 1.0–1.8%) after laparoscopic and 3.8% (95% CI 3.1–4.4%) after open surgery. However, there were differences in both the type and indications for surgery [1]. In a recent meta-analysis of SBO after colorectal operations, the incidence of ASBO after laparoscopic surgery was somewhat lower than after open colorectal procedures (OR 0.62, 95% CI 0.54 to 0.72). However, no significant difference was found in the three randomized trials included in this review (OR 0.50, 95% CI 0.20 to 1.2) [39]. In summary, there is some evidence that the incidence of ASBO is lower after laparoscopy. However, the effect seems modest when correcting for type and indication of surgery. Thus, performing (colorectal) surgery by laparoscopy is not a complete solution to preventing adhesive SBO.

Many other aspects of surgical technique have been associated with adhesion formation, although there are little or no epidemiological data concerning their impact on the incidence of ASBO. Nevertheless, a number of important risk factors for aggravated adhesion formation are worth considering. One of the most important risk factors is the foreign body reaction, for example as seen with starch-powdered gloves, and meshes used for abdominal wall reconstruction [40, 41]. The choice of energy device might also impact adhesion formation. Peritoneal injury is lower in bipolar electrocautery and ultrasonic devices as compared to monopolar electrocautery [42, 43]. Animal data suggest that both systemic and intraperitoneal application of antibiotics, and metronidazole in particular, can reduce adhesion formation in septic conditions [44, 45].

#### Adhesion barriers

Adhesion barriers are adjuvants for peritoneal administration that can effectively reduce adhesion formation. Adhesion barriers are produced in several forms: solid membranes, gels, and liquids. The concept behind barriers is that they do not actively interfere with inflammation and wound healing. Rather, they act as a spacer which separates injured surfaces of the peritoneum, allowing these surfaces to heal without forming fibrinous attachments which eventually lead to adhesions. In order to accomplish this task, such barriers should ideally be inert to the human immune system and be slowly degradable.

There is moderate evidence that a hyaluronate carboxymethylcellulose adhesion barrier can reduce the incidence of reoperations for ASBO in colorectal surgery. In three trials involving 1132 patients undergoing colorectal surgery, hyaluronate carboxymethylcellulose reduced the incidence of reoperations for adhesive small bowel obstruction (RR 0.49, 95% CI 0.28–0.88) [46–48]. The use of such barriers seems cost-effective in open colorectal surgery [49]. An overview of common used adhesion barriers and their efficacy is found in Table 4.

**Table 4:** Overview of most common applied adhesion barriers and their impact on adhesion formation and incidence of ASBO

Barrier	Marketed as	Comments
Hyaluronate carboxymethylcellulose	Seprafilm®	Solid barrier most suitable for open surgery although laparoscopic placement has been described Studies in both general surgery and gynecological procedures Reduces adhesion formation, as well as the risk for reoperations for adhesive small bowel obstruction (relative risk 0.49, 95% CI 0.28–0.88)
Oxidized regenerated cellulose	Interceed®	Solid barrier most suitable for open surgery Only studied in gynecological procedures Reduces incidence of adhesion formation relative risk 0.51, 95% CI 0.31–0.86 No studies available on subsequent risk of ASBO This workgroup does not recommend the use of this barrier to prevent ASBO in general surgery
Icodextrin	Adept®	Liquid barrier, easy to apply in both open and laparoscopic surgery Good safety record in both general surgery and gynecological surgery Reduces recurrence of ASBO following surgery for ASBO in one trial (relative risk 0.20, 95% CI 0.04–0.88)
Polyethylene glycol	Sprayshield®/Spraygel®	Gel barrier, easy to apply in both open and laparoscopic surgery Reduces adhesion score in both general surgery and gynecological trials Relative few and small studies, impact on long-term adhesion-related complications not described

Adapted from [52]

#### Secondary prevention

Adhesion barriers might also be useful to prevent recurrence after surgical treatment of ASBO. One randomized trial with an adhesion barrier included patients undergoing surgery for ASBO [20]. In this trial, patients were randomized to a liquid 4% icodextrin adhesion barrier or standard operative treatment without an adhesion barrier. The ASBO recurrence rate was 2.19% (2/91) in the icodextrin groups versus 11.11% (10/90) in the control group after a mean follow-up period of 41.4 months (*p* < 0.05) [20]. In this trial, the barrier was applied in patients treated for ASBO by laparotomy. However, the icodextrin 4% adhesion barrier can also be administered in laparoscopic surgery. Other trials with icodextrin as an adhesion barrier indicated that it actually might not be the most potent barrier to prevent adhesion reformation, which is typically more challenging than prevention of de novo adhesions [50]. Favoring the use of icodextrin are its low costs and good safety record [51]. From the results of other trials, we suggest that a hyaluronate carboxymethylcellulose might be more efficacious, but this barrier is less practical in laparoscopic surgery [46–48, 52].

### Approach to the patient with ASBO

**Fig. 2:**
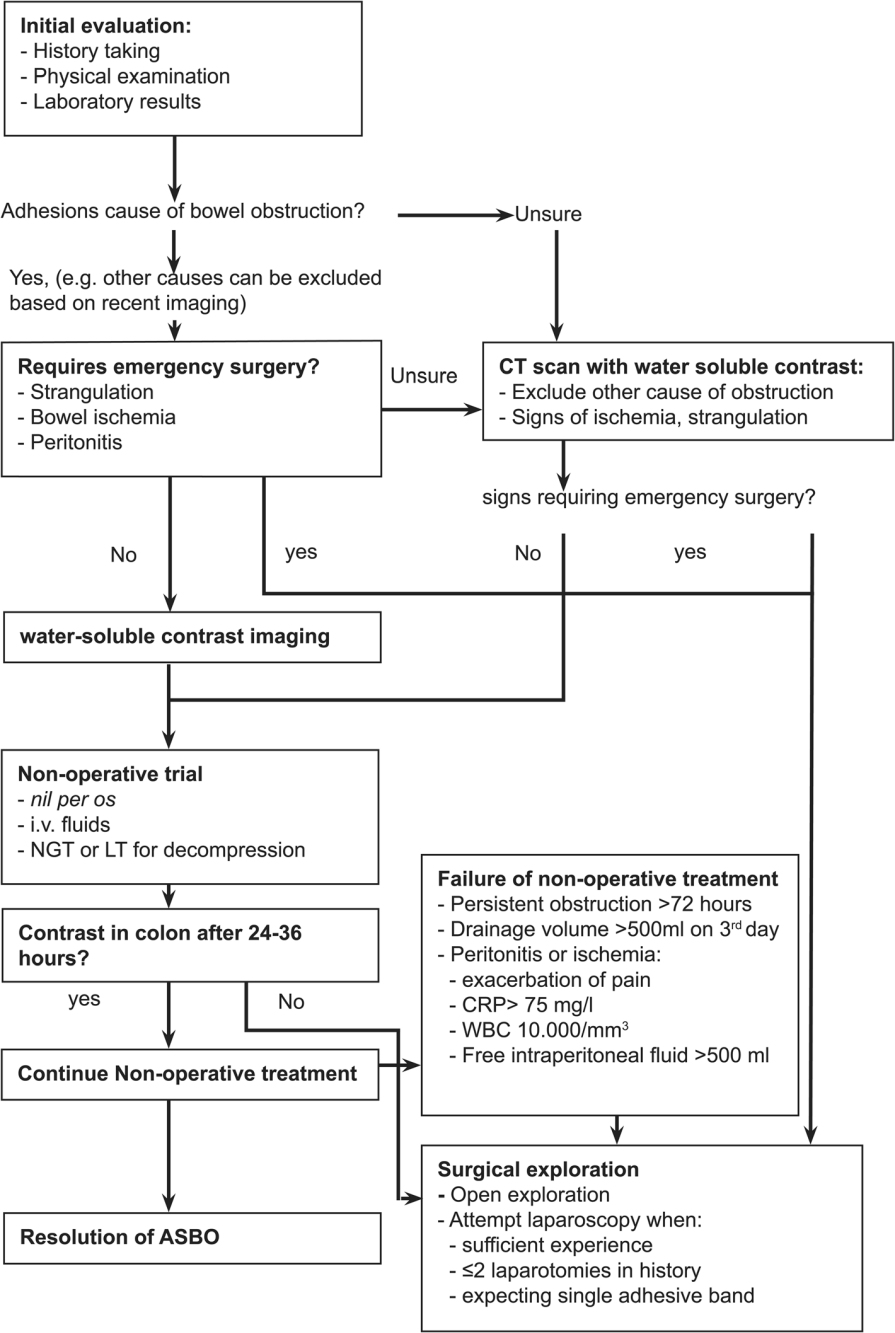
Algorithm to diagnosis and treatment of ASBO

An algorithm for the diagnostic and therapeutic approach to the patient with ASBO is presented in Fig. 2. The initial diagnosis of ASBO is of utmost importance. Failure to diagnose or having a delayed diagnosis represents 70% of malpractice claims in ASBO [53, 54].

The primary goals in the initial evaluation of patients in whom adhesive small bowel obstruction is suspected are:

- Differentiating between adhesive small bowel obstruction and other causes of bowel obstruction
- Assessing the need for urgent surgical exploration
- Identifying and preventing complications from bowel obstruction

#### History taking and physical examination

History taking in a patient suspected for ASBO includes assessment of potential causes of bowel obstruction (previous operations, radiotherapy) and nutritional status. Signs of dehydration should also be assessed. Traditionally, ASBO is clinically diagnosed in a patient with intermittent colicky abdominal pain, distention, and nausea (with or without vomiting), with or without absence of stools. Although diagnosis of small bowel obstruction is fairly certain in a patient in whom all of these symptoms are present, there are some specific pitfalls that can result in delayed or misdiagnosis of bowel obstruction upon initial presentation. In patients with incomplete obstruction, watery diarrhea may be present. The presence of watery diarrhea can cause an episode of ASBO to be mistaken for gastro-enteritis. Stools might also be present in patients with a relatively high obstruction who are admitted early after onset of symptoms. Moreover, not all of these symptoms may be present, especially in the elderly in whom pain is often less prominent [55, 56].

During physical examination, signs of peritonitis that might reveal strangulation or ischemia should be evaluated. Differential diagnostic considerations that can be assessed during physical examination include the presence of any abdominal wall or groin hernias. The evaluation of ASBO by history taking and physical examination has a low sensitivity for detecting bowel strangulation and ischemia. Sensitivity of physical examination for detection of strangulation is only 48%, even in experienced hands [57].

#### Laboratory tests

The minimum of laboratory tests include blood count, lactate, electrolytes, CRP, and BUN/creatinine. Laboratory values that might indicate peritonitis are a CRP > 75 and white blood cell count > 10.000/mm^3^, although sensitivity and specificity of these tests are relatively low [6, 57, 58]. Electrolytes are often disturbed in patients with a bowel obstruction; in particular, low values of potassium are frequently found and need to be corrected. BUN/creatinine needs to be assessed as patients with ASBO are frequently dehydrated which could result in acute kidney injury.

### Imaging studies

#### Plain X-rays

The value of plain X-rays complementary to physical examination is limited. In high-grade obstruction, a triad of multiple air-fluid levels, distention of small bowel loops, and absence of gas in the colon are pathognomonic for small bowel obstruction, but overall sensitivity and specificity of plain x-rays are low (sensitivity approximately 70%) [59, 60]. A large volume pneumoperitoneum secondary to bowel perforation in ASBO can also be detected on plain X-rays, preferably by an erect chest X-ray. Plain X-rays, however, do not detect the more early signs of peritonitis or strangulation [59–61]. Furthermore, a plain abdominal X-ray does not provide anatomical information that helps differentiate between the various causes of bowel obstruction.

#### Water-soluble contrast studies

Several systematic reviews and meta-analyses have established the usefulness of water-soluble contrast agents in the diagnostic work-up of ASBO [62–64]. If the contrast has not reached the colon on an abdominal X-ray taken 24 h following administration of the contrast, this is highly indicative of failure of non-operative management. Multiple studies have shown that the use of water-soluble contrast agents accurately predicts the need for surgery and reduces hospital stay [62, 63]. Some authors also suggest that water-soluble contrast studies reduce the need for surgery, which is attributed to an active therapeutic role of the contrast [62, 63].

#### CT scans

Current helical CT scans not only have good test characteristics for diagnosing small bowel obstruction but also have approximately 90% accuracy in predicting strangulation and the need for urgent surgery [37, 60, 65–68]. Diagnostic value of CT scan can be enhanced with the use of water-soluble contract. As with water-soluble contrast studies, progress of the contrast can be evaluated by X-ray at 24 h after CT scan.

Although adhesions are not directly visible even on CT scan, a CT scan can differentiate accurately between different causes of bowel obstruction by excluding other causes. The workgroup therefore considers CT scan to be the preferred imaging technique if there is any doubt about the diagnosis of ASBO, and to assess the need for urgent surgery.

A CT scan should help to differentiate between a complete obstruction of the bowel and help facilitate the decision for a trial of non-operative management versus a decision to proceed to surgery. It may also help to define the location of the obstruction (e.g., high in the jejunum or deep in the pelvis). Signs of a closed loop, bowel ischemia, and free fluid are signs that suggest the need for surgery without delay. In addition, radiological and clinical scores can be used to predict the need for surgery as described above [37, 38].

#### Ultrasound and MRI

Although the working group considered CT scan to be the preferred technique for diagnosis of ASBO, ultrasound and MRI might be useful in specific situations. Ultrasound is operator dependent but in experienced hands can provide more information than plain X-rays, and is also available in most low income settings. Apart from distension of bowel loops, ultrasound enables detection of free fluid (that might indicate the need for urgent surgery) and assessment of the degree of shock in dehydrated patients [61, 69]. Ultrasound can also be of value in situations in which exposure to radiation is undesirable, such as in pregnant patients. In these cases, ultrasound might be complemented with MRI for more anatomical information if the diagnosis of bowel obstruction is confirmed [70].

#### Diagnosis: summary

Recommendations can be found in Table 5. In summary, CT scan with oral water-soluble contrast is the preferred technique of imaging in the initial evaluation. Progress of the contrast should be monitored after 24 h of non-operative treatment by X-ray. If the diagnosis of ASBO is certain (e.g., because other causes have been excluded with recent imaging), and there are no signs that immediate surgery might be warranted, only a water-soluble contrast study is considered sufficient. Ultrasound and MRI can be useful in specific situations, such as pregnancy or (in low income countries) when CT scan is unavailable.

**Table 5:** Overview of conclusions and recommendation

Level A	Adhesive small bowel obstruction is a leading cause of morbidity, deaths, and healthcare expenditures in emergency surgery. *A2 Scott 2016*; *NELA project team 2016*
Level B	Adhesive small bowel obstruction causes high morbidity, with average hospital stay of 8 days and 3% in-hospital mortality per episode. Recurrence of adhesive small bowel obstruction is high. Risk for adhesive small bowel obstruction may be somewhat lower after laparoscopic compared to open colorectal surgery, but that results could not be confirmed in randomized trials. *A2 ten Broek 2013*; *Yamada 2016*; *B Krielen 2016*; *Foster 2006*
Level IB	Laparoscopic surgery reduces adhesion formation and might reduce subsequent incidence of ASBO. *B Lundorff 1992*; *ten Broek 2013*; *Yamada 2016*
Level IA	Hyaluronate carboxymethylcellulose reduces adhesion formation and the risk of subsequent reoperations of adhesive SBO. The use of this barrier seems cost-effective in open colorectal surgery. *A1 ten Broek 2014*; *A2 Fazio 2006*; *Park 2009*; *Kusunoki 205*
Level IIC	In the absence of signs that require emergent surgical exploration (i.e., peritonitis, strangulation, or bowel ischemia), non-operative management is the treatment strategy of choice. *C Fevang 2002*; *Fevang 2004*; *Ten Broek 2013*; *Jeppesen 2016*
Level IIB	A trial of non-operative management can be continued safely for 72 h. *B Keenan 2014*; *Sakakibara 2007*
Level IID	Initial evaluation should be complemented with assessment of nutritional status and laboratory tests evaluating at least blood count, lactate, electrolytes, and BUN/Creat *Expert opinion*
Level IIC	Plain X-rays have only limited value in the work-up of patients with small bowel obstruction and are not recommended. *B Maglinte 1996*
Level IB	Optimal diagnostic work-up should include CT scan in the assessment and water soluble oral contrast. In the absence of the need to perform immediate surgery, a follow-up abdominal X-ray should be made after 24 h. If the contrast has reach the colon, this is indicative for resolution of the bowel obstruction. *A2 Ceresoli 2016*; *Branco 2010*; *Abbas 2005*; *B Goussous 2013*; *Zielinski 2011*; *Zielinski 2010*; *Daneshmat 1999*; *Makita 1999*; *Zalcman 2000*
Level IIC	Long trilumen naso-intestinal tubes are more efficacious than naso-gastric tubes in non-operative management, but require endoscopic placement. *A2 Chen2012*
Level IIC	Laparoscopic adhesiolyis might reduce morbidity in selected cases of ASBO that require surgery. Results of a randomized trial are awaited. *B Sajid 2016*; *Farinella 2009*; *Sallinen 2014*
Level IIB	Adhesion barriers reduce the risk of recurrence for ASBO following operative treatment. *A2 Catena 2012*
Level IIC	Younger patients, and pediatric patients in particular, have higher lifetime risk of developing adhesion-related complications and might therefore benefit most from adhesion prevention. *A1 ten Broek 2013*; *A2 Strik 2016*; *B Fredriksson 2016*
Level C	More research is needed to the impact of comorbidities in elderly patients on optimal management of adhesive small bowel obstruction. Patients with diabetes might require more early operative intervention. *B Karamanos 2016*

### Management

#### Initial decision making

Non-operative management should always be tried in patients with adhesive small bowel obstruction, unless there are signs of peritonitis, strangulation, or bowel ischemia [71]. Although the risk of recurrence is slightly lower after operative treatment, this is not a reason to opt for a primary surgical approach. Morbidity from emergency surgical exploration is high; there is a considerable risk for bowel injury, and surgical treatment may significantly reduce post-operative quality of life [1, 72–74].

#### Non-operative management

The cornerstone of non-operative management is *nil* per os and decompression using a naso-gastric tube or long intestinal tube. Non-operative management is effective in approximately 70–90% of patients with ASBO [1, 75, 76]. There has been some debate in the literature over the use of long intestinal tubes or naso-gastric tubes. In an older trial, no significant difference in failure rates was found between naso-gastric tubes and long intestinal tubes [77]. In a more recent trial, 186 patients were randomized between a newly designed trilumen long tube and a naso-gastric tube. Long tubes seemed more effective in this trial with a failure rate of 10.4% in this group compared with 53.3% in the naso-gastric tube group [78]. Results from this trial should be interpreted with care, because the failure rate of naso-gastric tube compression is much higher than would be expected from other literature. Moreover, a drawback of trilumen tubes is the need for endoscopic placement. Non-operative management should further include fluid resuscitation, correction of electrolyte disturbances, nutritional support, and prevention of aspiration.

Duration of the period in which non-operative management can be tried is subject to debate. Several retrospective series and databases have shown that delays in surgery increase morbidity and mortality [30, 71, 79, 80]. Evidence for the optimal duration of non-operative treatment is absent, but most authors and the panel consider a 72-h period as safe and appropriate [11, 58, 76, 79, 80]. Continuing non-operative treatment for more than 72 h in cases with persistent high output from a decompression tube, but no other signs of clinical deterioration, however, remains subject to debate. Common medical complications in patients with small bowel obstruction are dehydration with kidney injury, electrolyte disturbances, malnutrition, and aspiration.

#### Non-operative management: summary

The panel recommends a trial of non-operative management in all patients with ASBO, unless there are signs of peritonitis, strangulation, or bowel ischemia. Evidence for the optimal duration of non-operative is absent, but most authors and the panel consider a 72-h period as safe and appropriate. Further recommendations are found in Table 5.

#### Operative treatment

Historically, abdominal exploration through laparotomy has been the standard treatment for adhesive small bowel obstruction. In recent years, however, laparoscopic surgery for ASBO has been introduced. The potential benefits of laparoscopy include less extensive adhesion (re)formation, earlier return of bowel movements, reduced post-operative pain, and shorter length of stay [81–83]. In a recent systematic review and meta-analysis of 14 non-randomized studies, laparoscopic adhesiolysis reduced risk of morbidity, in-hospital mortality, and surgical infections [84]. However, there also seems strong selection bias in these series allocating mainly the less severe cases to laparoscopy. In a questionnaire among surgeons, 60% of the respondents reported to have performed laparoscopic adhesiolysis for ASBO in their practice, but half of them in less than 15% of cases [11].

Although laparoscopy might provide some benefits to some patients for ASBO, surgeons should carefully select candidates for laparoscopic treatment. Laparoscopy in an abdomen with very distended loops of bowel and multiple complex adhesions could increase the risk of severe complications such as enterotomies and delayed diagnosis of perforations [85, 86]. Indeed, some authors have reported bowel injury in 6.3 to 26.9% of patients treated with laparoscopic adhesiolysis for ASBO [87–89]. In a recent population-based study, bowel resections were significantly more frequent in laparoscopic surgery. Incidence of bowel resection was 53.5 versus 43.4% in laparoscopic versus open procedures [90]. Farinella et al. reported that predictors for a successful laparoscopic treatment of ASBO are the following: ≤ 2 laparotomies in history, appendectomy as the operation in history, no previous median laparotomy incision, and a single adhesive band [91]. Laparoscopic adhesiolysis also seems more difficult in patients who have previously been treated by radiotherapy [92].

More compelling evidence on the role of laparoscopy in surgery for ASBO is from an ongoing randomized trial and is still awaited [93]. In this trial, strict inclusion and exclusion criteria have been used to select candidates in whom simple single band adhesions are expected.

#### Operative management: summary

Laparoscopic surgery has been introduced in recent years and might decrease morbidity in subgroups of patients undergoing surgery for ASBO. The risk of bowel injuries seems higher in laparoscopic surgery for ASBO. Therefore, careful selection of patients for laparoscopic surgery is required. Further recommendations are found in Table 5.

### Special patient groups

#### Young patients

The risk of adhesion-related complications is life-long. Although most small bowel obstructions will occur within the first 2 years after surgery, new cases continue to develop many years after the primary operation [1, 30, 72, 94, 95]. Also, the risk of requiring a future reoperation for unrelated causes is higher in younger patients [96]. Pediatric patients, who are at the extreme of young age, have a high risk for adhesion-related complications [1]. In a recent cohort of patients who underwent surgery at a pediatric age, the incidence of adhesive small bowel obstruction was 12.6% after a median follow-up of 14.7 years [29].

Young patients therefore might have the highest lifetime benefit from adhesion prevention [49]. No trials with adhesion barriers have been performed in pediatric surgery, but a recent cohort study in pediatric patients showed a significant reduction in ASBO with the use of a hyaluronate carboxymethylcellulose adhesion barrier [97]. After a follow-up of 24 months, 2.0% of pediatric patients operated with adhesion barrier versus 4.5% of patients operated on without adhesion barrier developed ASBO.

#### Elderly patients

In elderly patients, quality of life considerations are extremely important in decision making. Patients with a high frailty index have a prolonged recovery after a surgical procedure and may not be able to return to their previous functional state and quality of life [98, 99].

The principles of treatment for adhesive small bowel obstruction might interfere with comorbidities and medication in the elderly patients. There is a marked paucity of research on the consequences of stopping or withholding oral medications when a patient is put on *nil* per os for non-operative treatment of small bowel obstruction. A recent cohort showed that patients with diabetes might require earlier intervention although the level of evidence is rather low. Patients with diabetes were shown to suffer from a 7.5% incidence of acute kidney injury and 4.8% incidence of myocardial infarction if the operation was delayed more than 24 h [100]. The incidence of these complications was significantly higher when compared to diabetic patients that were operated within 24 h and non-diabetic patients with delayed operation.

#### Pregnancy

Small bowel obstruction in pregnancy is very rare but represents an important clinical challenge with significant risk of fetal loss. In a recent review, 46 cases of bowel obstruction during pregnancy were found in literature from case series and case reports [101]. Approximately half of cases were attributed to adhesions, most commonly from previous abdominal operations. Imaging studies performed to diagnose SBO in the case reports included ultrasound in ten cases (83%), abdominal X-ray in four patients (33%), MRI in four patients (33%), and a CT scan in three patients (25%). Strikingly, the failure rate of non-operative treatment in pregnant patients with ASBO was high. A total of 23 cases with ASBO were reported, in 17 of whom initial management was by a non-operative trial. Non-operative treatment failed in 16 cases (94%). Risk of fetal loss was 17% (*n* = 8) and risk of maternal death 2% (*n* = 1).

## Conclusions

The conclusions and recommendations of this guideline have been summarized in Table 5. ASBO is a common surgical emergency, causing high morbidity and even some mortality. Surgeons should be aware that the adhesions causing such bowel obstructions are typically the footprints of previous abdominal surgical procedures or disease. Part of the adhesion formation can be prevented by application of minimal invasive surgical techniques and the use of adhesion barriers. Most cases of ASBO can be treated non-operatively. If operative treatment is required, a laparoscopic approach might be beneficial for simple cases. However, there is a considerable risk for conversion to an open laparotomy and care needs to be taken not to make bowel injury.
